# Phacoviscocanalostomy *versus* phacotrabeculectomy to treat glaucoma associated with cataracts: a meta-analysis

**DOI:** 10.31744/einstein_journal/2025RW1045

**Published:** 2025-03-17

**Authors:** Dillan Cunha Amaral, Mário Luiz Ribeiro Monteiro, Denisse J. Mora-Paez, Ana Luiza Machado Ribeiro Pimentel, Matheus Mizerani Fernandes de Almeida, Jacqueline L. Chen, Raíza Jacometti, Milton Ruiz Alves, Jaime Guedes, Ricardo Noguera Louzada

**Affiliations:** 1 Universidade Federal do Rio de Janeiro Faculdade de Medicina Rio de Janeiro RJ Brazil Faculdade de Medicina, Universidade Federal do Rio de Janeiro, Rio de Janeiro, RJ, Brazil; 2 Universidade de São Paulo Faculdade de Medicina Divisão de Oftalmologia e Laboratório de Investigação em Oftalmologia (LIM-33) São Paulo SR Brazil Divisão de Oftalmologia e Laboratório de Investigação em Oftalmologia (LIM-33), Faculdade de Medicina, Universidade de São Paulo, São Paulo, SR Brazil; 3 Wills Eye Hospital Glaucoma Research Center Philadelphia PA USA Glaucoma Research Center, Wills Eye Hospital, Philadelphia, PA, USA; 4 Pontifícia Universidade Católica de Goiás Faculdade de Medicina Goiânia GO Brazil Faculdade de Medicina, Pontifícia Universidade Católica de Goiás, Goiânia, GO, Brazil; 5 Centro Universitário de Valença Faculdade de Medicina Valença RJ Brazil Faculdade de Medicina, Centro Universitário de Valença, Valença, RJ, Brazil; 6 Thomas Jefferson University Sidney Kimmel Medical College Philadelphia PA USA Sidney Kimmel Medical College, Thomas Jefferson University, Philadelphia, PA, USA

**Keywords:** Glaucoma, Phacoemulsification, Viscocanalostomy, Trabeculectomy, Cataract

## Abstract

**Objective:**

To compare the effectiveness and safety of phacoviscocanalostomy and phacotrabeculectomy in treating combined glaucoma and cataracts.

**Methods:**

A systematic review and meta-analysis were conducted following the PRISMA guidelines. The PubMed, Web of Science, Cochrane, and Embase databases were searched for randomized controlled trials or observational studies comparing phacotrabeculectomy to phacoviscocanalostomy in patients with glaucoma and cataracts. Statistical analysis was used to compare the efficacy (intraocular pressure reduction, mean deviation of the visual field, and failure rates) and safety (general complication rate and rates of hyphema, hypotony, perforation, and intraocular pressure spikes) between the two procedures.

**Results:**

The study included 331 eyes from one randomized controlled trial and two non-randomized controlled trials, with 154 undergoing phacoviscocanalostomy and 177 undergoing phacotrabeculectomy. The results indicated no significant differences in surgical failure rates, mean deviation of the visual field, and intraocular pressure at one, three, six, and twelve months between the phacoviscocanalostomy and phacotrabeculectomy groups. Furthermore, although the overall complication rate between the two procedures showed no difference, the rate of intraocular pressure spikes was higher in patients who underwent phacoviscocanalostomy.

**Conclusion:**

Phacotrabeculectomy and phacoviscocanalostomy are effective treatments for glaucoma and cataracts.

**Prospero database registration:** (www.crd.york.ac.uk/prospero) under ID CRD42024502391.

## INTRODUCTION

Glaucoma is a leading cause of blindness worldwide.^(1,2)^ In 2020, the number of people with glaucoma worldwide was estimated to be >70 million, and this number is expected to increase to >110 million by 2040.^(1)^ Owing to the growing number of patients with glaucoma, new surgical techniques are continuously being developed and improved. These innovations are crucial, because early glaucoma treatment can significantly delay vision loss.^(3)^

The only known adjustable risk factor for glaucoma is high intraocular pressure (IOP), and several therapies have been designed to lower IOP to prevent or minimize vision loss caused by high pressure. These therapies include medication, laser treatments, and surgical interventions.^(4,5,6,7)^ The gold standard surgical procedure for treating high pressure.^(6,8)^ A trabeculectomy is a filtering procedure involving the dissection of a partial-thickness scleral flap under the conjunctiva and Tenon's capsule, followed by paracentesis and complete sclerotomy to remove a portion of the sclera.^(9)^ Another surgical procedure widely used for the treatment of glaucoma is viscocanalostomy, which was described in 1999 by Stegmann and consists of identifying the Schlemm's canal under a scleral flap and then dilating it with viscoelasticity, promoting the opening of the drainage system of the eye.^(10,11,12)^

Cataracts are clouds of the lens that affect thousands of people, and approximately 12 million people worldwide are blind because of cataracts.^(13)^ Due to the aging population, the coexistence of cataracts and glaucoma may become more common in the elderly population.^(14)^ This is particularly challenging because each treatment can influence the progression of the other; cataract removal surgery alone can reduce IOP levels. In contrast, glaucoma surgery alone can accelerate cataract progression.^(15)^

Given the coexistence of these two conditions, several surgical modalities have been developed to combine techniques for treating glaucoma and cataracts in a single surgery.^(16)^ Among these modalities are phacoviscocanalostomy (Phaco-Visco) and phacotrabeculectomy (Phaco-Trab).^(17)^

## OBJECTIVE

This systematic review and meta-analysis aimed to compare the efficacy (measured using intraocular pressure reduction, visual field mean deviation, and success/failure rates) and safely (general complication rate and rates of hyphema, hypotony, perforation, or intraocular pressure spikes) of Phaco-Visco *versus* Phaco-Trab for combined glaucoma and cataract treatment.

## METHODS

This meta-analysis was performed according to the guidelines of the Declaration of Preferred Reporting Items for Systematic Reviews and Meta-Analyses (PRISMA) and the recommendations of the Cochrane Collaboration.^(18)^

### Eligibility criteria

Studies that met the following eligibility criteria were included: randomized control trials (RCTs) or observational studies; comparing Phaco-Visco to Phaco-Trab (ab interno trabeculectomy); patients aged ≥18 years with any type of glaucoma associated with cataract and no previous glaucoma surgery; follow-up time of at least one week; and reporting any of the clinical outcomes of interest. Studies with overlapping populations, case reports, animal studies, and *in vitro* experiments were excluded from the analysis.

### Data sourcing and search strategy

Two authors independently searched PubMed, Embase, Web of Science, and the Cochrane Library from inception to January 2024. Furthermore, the references in all included studies were manually searched for additional studies. Conflicts were resolved via consensus between authors. The following terms were used in the search: “phaco,” “phacoemulsification,” “cataract,” “phacoviscocanalostomy,” “phaco-viscocanalostomy,” “phacotrabeculectomy,” “phaco-trabeculectomy,” and “trabeculectomy.” Publication dates and language restrictions were not included in the electronic search of the studies.

### Study selection

The search results were imported into the reference management software, and duplicate records were excluded. Two authors independently applied the eligibility criteria to the titles and abstracts. The full texts of these titles and abstracts were reviewed to identify eligible studies. Disagreements were resolved by contacting the senior author.

### Data extraction

Two authors extracted the following data from the selected studies: country, study design, number of patients and eyes allocated to each arm, sex (male or female), follow-up time, and patient baseline characteristics. Pre-specified baseline characteristics, including country, type of study, number of eyes, mean age, male/female ratio, glaucoma type, mean preoperative IOP (mmHg), mean preoperative antiglaucoma medications, mean follow-up duration (months), mean preoperative best-corrected visual acuity (BCVA) (logMAR), mean postoperative BCVA (logMAR), and outcome data were recorded.

### Endpoints

The primary outcomes of interest were: IOP, success and failure rates, visual field-mean deviation, and general rates of complications. Specific analysis of adverse events examined the rates of hyphema, transient hypotony, perforation of the Descemet membrane, and IOP spikes. Kobayashi et al.^(17)^ defined overall success as achieving an IOP of 6-20mmHg and/or a 30% IOP reduction with or without a single topical agent. In contrast, Jiang et al.^(19)^ defined success as an IOP below 20mmHg and/or a 20% IOP reduction with or without a single topical agent. The other studies have not reported such data. Kobayashi et al^(17)^ defined failure as the need for a new filtering surgery, whereas Jiang et al.^(19)^ defined failure as the need for more than one topical agent and/or repeat surgery. The general complications considered in the analysis included hyphema, shallow anterior chamber, bleb leak, fibrin reaction, layered hyphema, Descemet's detachment/hemorrhage, lens malposition, perforation of Descemet's membrane, choroidal detachment, hypotensive maculopathy, IOP spike, peripheral anterior synechiae, posterior synechiae, failed bleb, bleb formation, rupture of the posterior lens capsule, vitreous loss, and Schlem's tube piercing.

### Statistical analysis

Treatment effects for binary endpoints were compared using pooled odds ratios (ORs) with 95% confidence intervals (95%CIs). Differences in continuous variables were compared using mean differences (MD). The Cochrane Q-test and I^2^ statistics assessed heterogeneity; p>0.10 and I^2^ >25% values were considered significant for heterogeneity. Statistical significance was defined as p<0.05. The Sidik-Jonkman estimator was used to calculate the tau^2^ variance between studies. In addition, a random-effects model was used for all pooled outcomes. Statistical analyses were performed using R software (version 4.2.3, R Foundation for Statistical Computing, Vienna, Austria).

## RESULTS

### Study selection and baseline characteristics

**Figure 1:**
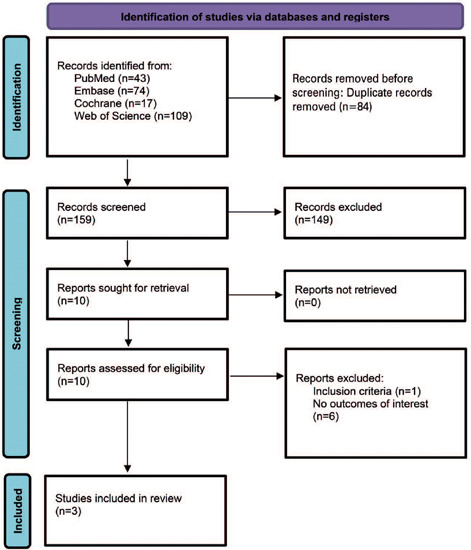
Study screening and selection

As detailed in figure 1, 243 articles were found: 43 in PubMed (Medline), 74 in Embase (Elsevier), 109 in Web of Science, and 17 in Cochrane databases. Of these, 84 were excluded as duplicates. After removing duplicate records and ineligible studies, ten studies remained, and seven of these met the inclusion and exclusion criteria. The final number of studies included one RCT^(17)^ and two non-randomized retrospective cohorts, with a total of 331 eyes.^(19,20)^ Of these eyes, 177 (53.5%) underwent Phaco-Trab, and 154 (46.5%) underwent Phaco-Visco. The study characteristics are shown in table 1. The intraoperative metabolites and postoperative interventions are shown in table 2.

**Table 1:** Baseline characteristics

Study				P-T								P-V							
	Country	Type of study	Eyes P-T/P-V	Mean age (yr)	Male: female	Glaucoma type	Mean preop IOP (mmHg)	Mean preop antiglaucoma medications	Mean follow-up (months)	BCVA preop (logMAR)	BCVA postop* (logMAR)	Mean age (yr)	Male: female	Glaucoma type	Mean preop IOP (mmHg)	Mean preop antiglaucoma medications	Mean follow-up (months)	BCVA preop (logMAR)	BCVA postop* (logMAR)
Yao et al.^(20)^	China	R	28/30	69.5 (±9.4)	12/15	28 POAG	24.5 (±5.2)	NA	6	NA	NA	69.4 (±10.1)	14/14	30 POAG	25 (±5.4)	NA	6	NA	NA
Kobayashi et al^(17)^	USA	RCT	20/20	71 (±7.7)	10/10	40 POAG	23.7 (±2.6)	2.6 (±0.9)	33.4 (±20.9)	0.643 (±0.288)	0.0430± (0.062)	71.5 (±8.9)	11/9	40 POAG	24.0 (±2.0)	2.8 (±0.8)	30.3 (±15.8)	0.678 (±0.296)	0.026 (±0.058)
Jiang etal.^(19)^	England	R	129/104	71 (±10.3)	71/58	105 POAG + 24 PACG	23.4 (±8.3)	2.5 (±0.9)	23.2 (±11.5)	0.23 (±0.105)	0.19 (±0.15)	67.9 (±8.8)	46/58	88 POAG + 16 PACG	20.2 (±4.2)	2.3 (±0.9) (	26.5 ±13.3)	0.32 ±0.24)	0.14 (±0.12)

^#^1 year of follow-up.

R: retrospective; RCT: randomized clinical trial; P-V: phacoviscocanalostomy; P-T: phacotrabeculectomy; POAG: primary open-angle glaucoma; PACG: primary angle-closure glaucoma; IOP: intraocular pressure; BCVA: best-corrected visual acuity; Yr: years; NA: not applicable.

**Table 2:** Intra- and postoperative characteristics

Study	Country	Type of study	Eyes P-T/P-V	P-T		P-V	
				Intraoperative metabolites	Postoperative interventions	Intraoperative metabolites	Postoperative interventions
Yao et al.^(20)^	China	R	28/30	NA	NA	NA	NA
Kobayashi et al^(17)^	USA	RCT	20/20	MMC 0.04% for 3 min	Laser suture lysis was performed if the bleb was flat or the IOP was not low enough	NA	Goniopuncture was performed after surgery if the surgeon believed that the IOP was not low enough
Jiang et al.^(19)^	England	R	129/104	MMC (0.2mg/mL) for 3 min	Laser suture lysis was performed as necessitated by IOP and bleb condition. Subconjunctival needle revision with 5-fluororacil was administered to eight patients	MMC(0.2mg/mL) for 3 min	Laser suture lysis was performed as necessitated by IOP and bleb condition. Four patients required YAG laser goniopuncture following VC surgery. Subconjunctival needle revision with 5-fluororacil was administered to four patients

R: retrospective; RCT: randomized clinical trial; P-V: phacoviscocanalostomy; P-T: phacotrabeculectomy; MMC: mitomycin C; IOP: intraocular pressure; NA: not applicable.

### Pooled analysis of all studies

#### Intraocular pressure

**Figure 2:**
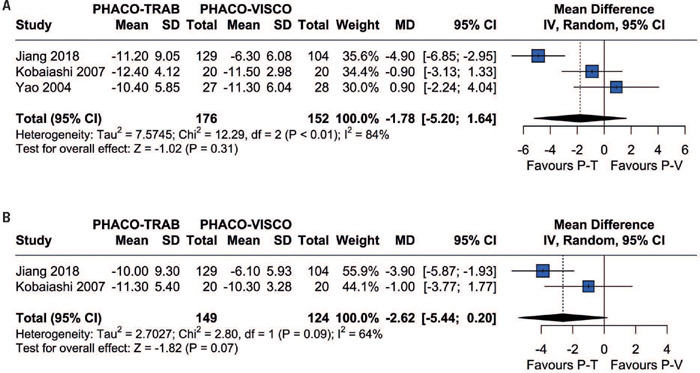
Forest plot of intraocular pressure at (A) one month and (B) three months

**Figure 3:**
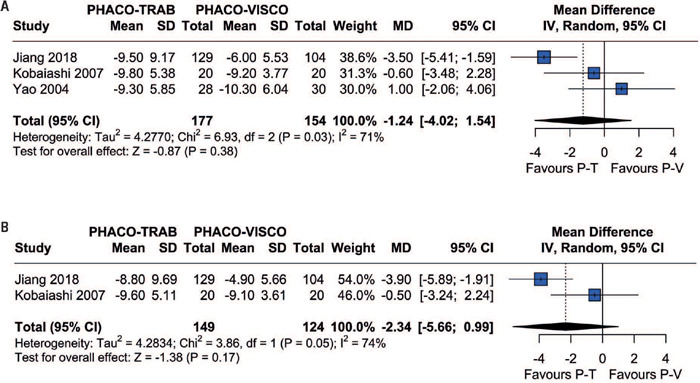
Forest plot of intraocular pressure at (A) six and (B) twelve months

The differences in IOP between the two procedures were analyzed at one, three, six and twelve months postoperatively. The included studies reported the incidence of IOP at one and six months. Pooled analysis from these studies revealed no significant differences in IOP lowering between patients in the Phaco-Trab and Phaco-Visco Groups at one (MD=-1.78; 95%CI=-5.20-1.64; p=0.31; I^2^=84%; Figure 2A) or six months (MD= -1.24; 95%CI=-4.02-1.54; p=0.38; P=71%; Figure 3A). In addition, three studies reported the IOP at three and twelve months. Analysis of these studies also revealed no significant differences between patients in the Phaco-Trab and Phaco-Visco Groups at three (MD= -2.62; 95%CI=-5.44-0.20; p=0.07; I^2^=64%; Figure 2B) and twelve months (MD=-2.34; 95%CI=-5.66-0.99; p=0.17; F=74%; Figure 3B).

#### Mean deviation of visual field

**Figure 4:**
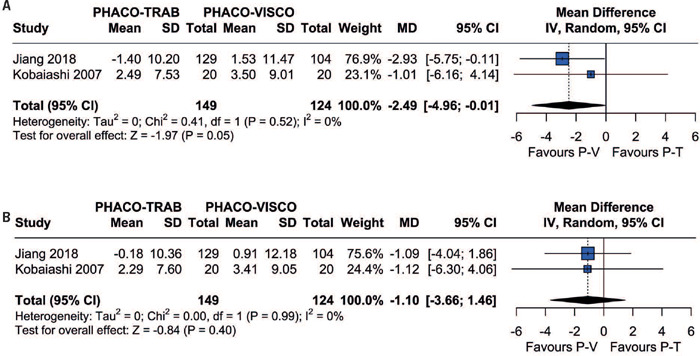
Forest plot of mean deviation at (A) six and (B) twelve months

Two of the included studies reported the mean deviation (visual field) after six and twelve months. These studies revealed no significant difference between patients in the Phaco-Trab and Phaco-Visco Groups at six (MD=-2.49; 95%CI=-4.96- -0.01; p=0.05; I^2^=0%; Figure 4A) and twelve months (MD=-1.10; 95%CI=-3.66-1.46; p=0.40; I^2^=0%; Figure 4B).

#### Success and failure rates

**Figure 5:**
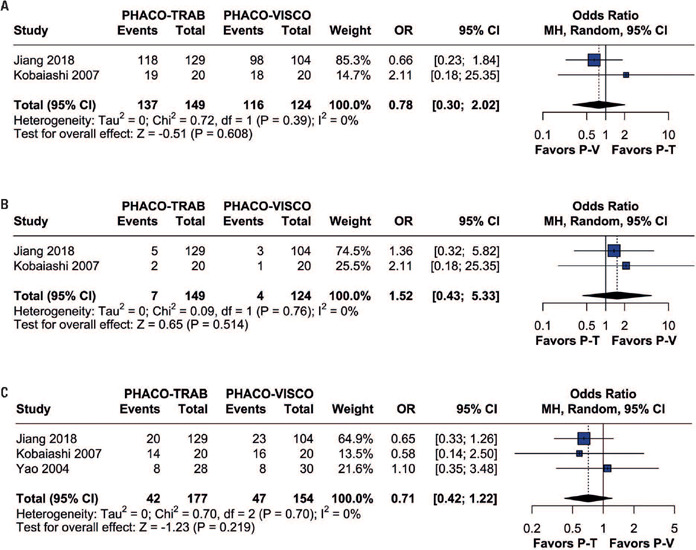
Forest plots of (A) overall success rates, (B) failure rates, and (C) general complications

The pooled results revealed no significant difference in success (MD=0.78; 95%CI=0.30-2.02; p=0.608; I^2^=0%; Figure 5A) and failure rates (MD=1.52; 95%CI=0.43 -5.33; p=0.514; I^2^=0%; Figure 5B) between patients in the Phaco-Trab and Phaco-Visco Groups.

#### Complication rates

**Figure 6:**
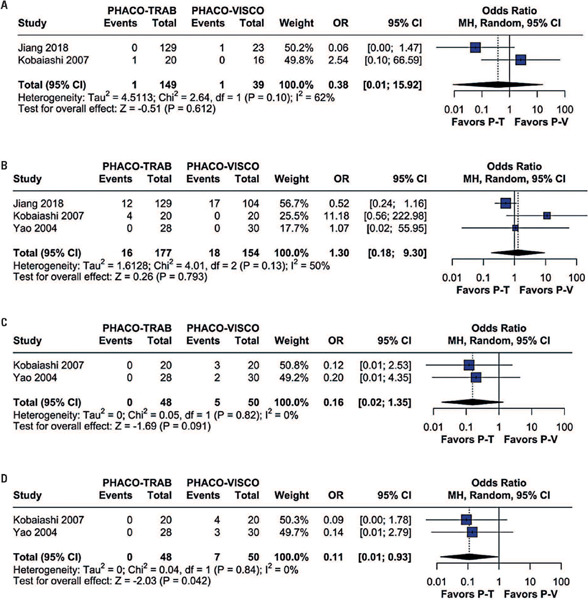
Forest plots of (A) hyphema, (B) transient hypotony, (C) perforation of Descemet's membrane, and (D) IOP spikes

The pooled results revealed no significant differences in general complications between patients in the Phaco-Trab and Phaco-Visco Groups (OR=0.71; 95%CI=0.42-1.22; p=0.219; I^2^=0%; Figure 5C). In addition to the general complication rate, specific complication rates for hyphemas, perforation of the Descemet membrane, IOP spikes, and transient hypotony were analyzed. There was no significant difference between patients in Phaco-Trab and Phaco-Visco Groups for hyphema (OR=0.38; 95%CI=0.01-15.92; p=0.612; I^2^=62%; Figure 6A), transient hypotony (OR=1.30;95%CI=0.18-9.30; p=0.793; I^2^=50%; Figure 6B), or perforation of Descemet's membrane (OR=0.16; 95%CI=0.02-1.35; p=0.091; I^2^=0%; Figure 6C). However, IOP spikes were significantly fewer in the Phaco-Trab Group compared to the Phaco-Visco Group (OR=0.11; 95%CI=0.01-0.93; p=0.042; I^2^=0%; Figure 6D).

## DISCUSSION

This meta-analysis of three studies comprising 331 eyes analyzed IOP reduction, mean deviation of the visual field, and complications of Phaco-Trab compared to Phaco-Visco. We found no significant difference in IOP reduction between the two surgical approaches at any time from one to twelve months postoperatively. Similarly, no significant differences were detected in the mean deviation of the visual field at six or twelve months postoperatively. In addition, the general complication rate and specific complication rates of hyphema, transient hypotony, or perforation showed no differences.

Our findings add to the literature on the outcomes of Phaco-Visco and Phaco-Trab. Although earlier studies reported lower success rates for singular viscocanalostomy compared to trabeculectomy,^(21,22)^ our findings indicate no significant difference between combined Phaco-Visco and Phaco-Trab. Furthermore, prior research has revealed that combining procedures results in a more pronounced hypotensive effect than viscocanalostomy alone.^(23,24)^

This meta-analysis revealed no significant difference in IOP reduction between the two groups at one, three, six, and twelve months after surgery. Since evaluating changes in the visual field of patients with glaucoma is crucial for monitoring vision loss, we also examined the mean deviation of the visual field.^(25,26)^ No significant differences were found between the two groups at six (MD=-2.49; p=0.05) and twelve months of surgery (MD=-3.66; p=0.40) after surgery. These results show that Phaco-Trab and Phaco-Visco maintained similar visual field progression patterns. Future studies should explore alternative testing strategies, such as frequency-doubling technology perimetry or short-wavelength automated perimetry, to complement the evaluation of visual field impact.^(26,27)^

A previous investigation associated primary viscocanalostomies with a lower complication rate than trabeculectomies.^(28)^ However, this meta-analysis did not reveal statistically significant differences in general complications between the two groups when combined with phacoemulsification (OR=0.71; p=0.219). However, the Phaco-Trab Group exhibited a lower rate of IOP spikes. Notably, the use of mitomycin C in two of the included studies may have resulted in late complications related to blebs, such as endophthalmitis and blebitis, as noted in previous studies.^(29,30,31,32)^

Although this study did not examine other factors, such as the learning curve and financial costs associated with these procedures, these factors should be considered when recommending treatment to patients. Thus, future research should analyze how the cost of the procedure may affect the recipients of each type of surgery and whether demographic factors or social determinants of health are associated with the outcomes of interest.

### Limitations

The relatively small number of RCTs constrained our analysis, because research in this field is limited. In addition, one of the studies included in the analysis had a maximum follow-up period of six months. Thus, the long-term effects of both procedures on glaucoma progression could not be assessed. The diverse glaucoma types included a lack of standardization in grouping, ethnic disparities, and an absence of standardized surgical techniques, which further contributed to the study's limitations. The absence of a standardized protocol for discontinuing preoperative glaucoma medications also introduced variability. However, to address these heterogeneity challenges, particularly when presenting general complications and IOP measurements at various postoperative intervals, we employed a random effects model to decrease the heterogeneity effect. Furthermore, the variation in treatment success and failure across studies introduced an additional layer of complexity to the analysis.

## CONCLUSION

Both Phaco-Trab and Phaco-Visco are effective treatments for glaucoma and cataracts. Our study found no significant differences in intraocular pressure reduction, mean deviation (visual field), and general complications between the two procedures. In addition, our study was limited by the small number of studies and relatively short follow-up periods. Therefore, future efforts should focus on large randomized long-term studies to validate these results.
